# Strengthening Copper Nano-Solder Pastes with Group IV 2D Materials: A Molecular Dynamics Insight

**DOI:** 10.3390/ma19071418

**Published:** 2026-04-02

**Authors:** Xuezhi Zhang, Jian Gao, Lanyu Zhang

**Affiliations:** State Key Laboratory of Precision Electronic Manufacturing Technology and Equipment, School of Electromechanical Engineering, Guangdong University of Technology, Guangzhou 510006, China; xuezhimail@163.com (X.Z.); lanyuzhang@gdut.edu.cn (L.Z.)

**Keywords:** copper nanoparticles, two-dimensional materials, composite paste, molecular dynamics simulation

## Abstract

This study investigates the effects of three group IV two-dimensional (2D) materials (graphene, silicene, and germanene) on the sintering process and tensile properties of copper nanoparticle pastes for electronic packaging. Using atomic-scale simulations, we constructed models of pure copper and composite pastes, tracking particle rearrangement, neck formation, and pore closure under identical sintering conditions, followed by uniaxial tensile testing. All composites formed continuous copper networks, with densification rates increasing in the order: graphene < silicene < germanene. The yield strength of the pure copper paste was 2.41 GPa and increased to 2.96, 4.39, and 5.46 GPa with graphene, silicene, and germanene, respectively, corresponding to gains of about 23%, 82%, and 127% relative to pure copper. Increasing the sintering temperature led to a monotonic increase in the tensile strength of the germanene composite, with the highest value being obtained at 650 K. Dislocation and stress field analyses revealed that silicene and germanene strengthen the material by promoting pronounced plastic accommodation in neck regions, whereas graphene mainly redistributes strain along the interfaces and produces a more moderate increase in strength. These findings demonstrate that the strength and deformation mode of copper nano-solder joints can be effectively tuned by selecting the type of 2D filler and optimizing the sintering temperature.

## 1. Introduction

Recent advances in three-dimensional (3D) integration, system-in-package, and heterogeneous chip integration have established these technologies as core approaches for enhancing computing density and enabling device miniaturization [1,2,3]. These packaging schemes universally rely on high-density, fine-pitch electrical interconnects, whose electrical and mechanical reliability is largely determined by the solder materials and their interfacial behavior [4,5]. Although traditional tin–lead solders offer excellent overall performance, their use has been strictly regulated due to environmental and human health concerns, driving the rapid development of lead-free solder systems [6,7]. Alloy solders such as Sn-Ag and Sn-Ag-Cu are now widely used in industry. However, as interconnect dimensions continue to shrink and operating temperatures increase, these alloys are proving vulnerable to limitations such as coarsening of intermetallic compounds, interfacial embrittlement, and thermomechanical failure [8,9].

Solder paste systems based on copper nanoparticles offer a promising material pathway to address the aforementioned challenges [10]. Meanwhile, bulk copper possesses a high melting point (1085 °C), which, at the nanoscale, is accompanied by a significant increase in surface energy, enabling the formation of continuous and dense metallic interconnection layers at relatively low sintering temperatures. Furthermore, these sintered copper structures maintain excellent thermal stability and creep resistance within typical device operating temperature ranges [11]. Compared to tin-based alloys, copper exhibits superior electrical and thermal conductivity, enabling a reduction in interconnection resistance and junction temperature rise. Its advantages also include remarkable resistance to electromigration, relatively low cost, and compatibility with silicon process flows [12]. Moreover, substantial experimental work has demonstrated that by optimizing the copper particle size distribution, organic formulation, and applied pressure conditions, high-strength Cu-Cu sintered joints can be achieved within a temperature range of 200–400 °C, with shear strengths that can approach or even surpass those of certain traditional solder systems [13,14].

Based on copper nanoparticle solder paste systems, the incorporation of suitable two-dimensional fillers has emerged as an effective strategy for regulating the sintered microstructure and mechanical performance [15]. Graphene, silicene, and germanene are particularly advantageous in this context, because their atomically thin geometry and high interfacial area favor intimate contact with Cu nanoparticles, thereby facilitating particle bridging, stress transfer, and local structural regulation during sintering and deformation [16,17,18]. Among them, silicene and germanene exhibit larger lattice constants, lower in-plane stiffness, and intrinsically buckled atomic configurations compared with graphene [19]. These structural characteristics provide greater interfacial conformability to evolving Cu nanoparticle surfaces, enhance local atomic accommodation during neck growth and pore shrinkage, and promote more efficient stress redistribution under mechanical loading. Such features are especially beneficial for nano-solder systems, where densification kinetics and joint integrity are governed by atomic rearrangement in highly confined interparticle regions. In addition, silicene and germanene offer clearer mechanistic advantages over many other emerging two-dimensional materials for metallic solder systems. Their monoelemental structure reduces compositional complexity and avoids additional interference arising from polar bonding, multicomponent chemistry, or surface terminations, which are commonly encountered in transition-metal dichalcogenides, hexagonal boron nitride, and MXenes [20,21,22]. As a result, the influence of intrinsic structural factors such as compliance, buckling, and surface corrugation on sintering behavior and mechanical strengthening can be evaluated more directly. Although graphene has been extensively studied as a reinforcing phase in tin-based and copper-based solders [16], the roles of silicene and germanene in copper nano-solder pastes remain largely unexplored, and comparative investigations under unified conditions are still very limited.

In realistic synthesis, impurities and defect-related non-ideal features are often unavoidable in two-dimensional materials and may affect local bonding, interfacial contact, and mechanical response. Nevertheless, under relatively low impurity levels, such effects are not expected to alter the intrinsic comparative trend dominated by filler structure, and the present idealized models therefore still provide a useful baseline for Cu nano-solder systems [23,24,25].

On the other hand, the processes of solder paste sintering and failure involve particle contact, atomic diffusion, pore shrinkage, and the initiation and propagation of dislocations and cracks, which are difficult to fully characterize solely through experimental techniques [26,27]. Molecular dynamics simulations, capable of tracking particle motion and local structural evolution at the atomic scale and quantifying diffusion coefficients, lattice distortions, and dislocation densities, are thus widely employed to investigate the sintering behavior and mechanical responses of nano-metallic systems [28]. Existing simulation work on copper nanoparticle–graphene composite systems has analyzed structural rearrangement in the neck region during sintering and the nucleation and transmission paths of dislocations under tensile loading, indicating that graphene influences overall strength by altering the stress distribution and dislocation slip mechanisms in the particle contact areas [29]. Other studies have examined the sintering process of copper nanoparticles on graphene coatings or substrates, revealing that interfacial interactions and particle size influence atomic diffusion rates and densification kinetics [30]. However, these findings predominantly revolve around carbon-based 2D phases; a systematic theoretical understanding of the effects of silicene and germanene introduction into copper nano-solder pastes on sintered density, dislocation behavior, and fracture modes remains lacking [31].

Based on the aforementioned background, this study employs classical molecular dynamics (MD) simulations to construct models of pure copper nanoparticle solder paste, as well as composite paste models doped with graphene, silicene, and germanene, designated as Cu-C, Cu-Si, and Cu-Ge, respectively. Under identical sintering conditions, we compare and analyze the particle rearrangement, neck formation, and pore shrinkage in each system during the heating and pressure-holding processes. The changes in atomic diffusion intensity and local order are characterized using mean squared displacement (MSD) and radial distribution function (RDF). Subsequently, uniaxial tensile loading is applied to the sintered structures at 300 K to obtain the stress–strain responses of the pure copper and the three composite pastes. Combined with dislocation analysis (DXA) and atomic stress distribution, the distinct effects of these group IV 2D materials (graphene, silicene, and germanene) on the mechanical properties of the copper nano-solder paste are investigated. By comparing the differences between the three 2D fillers and the pure copper system within a unified simulation framework, this work aims to elucidate, on the atomic scale, the governing principles of the group IV 2D material family in modulating the sintered microstructure and mechanical behavior of copper nano-solder pastes, thereby providing insights for the compositional design of highly reliable copper-based nano-solder pastes.

## 2. Molecular Dynamics Models and Simulations

This study conducted molecular dynamics (MD) simulations using the LAMMPS platform, with periodic boundary conditions being applied in all three directions. Temperature was controlled by a Nosé–Hoover thermostat, pressure was regulated using an isotropic barostat, and the time step was set to 1 fs. All trajectory data were visualized and post-processed using OVITO (Open visualization tool, version 3.13.0; available from https://www.ovito.org/). Simulations employed the metal unit style, ensuring consistency in the dimensions of stress, energy, and time with common scales used for metallic materials.

**Figure 1:**
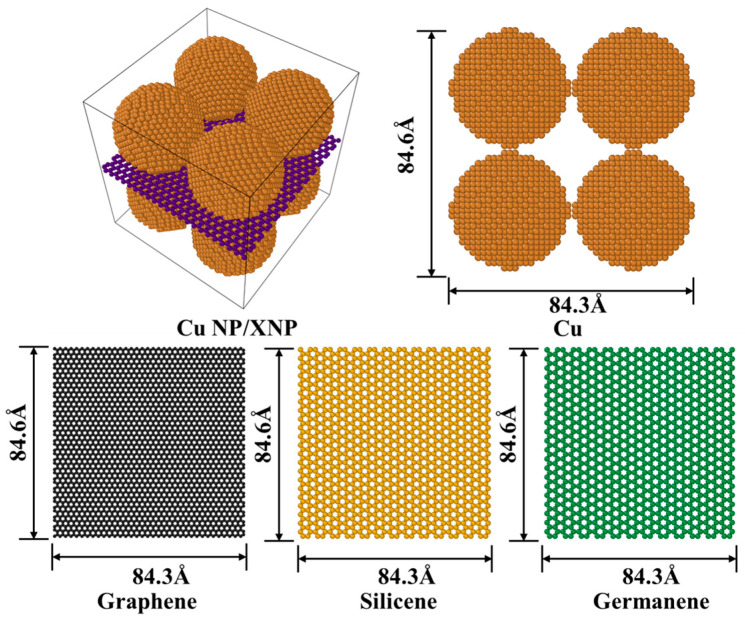
Initial configurations of the pure Cu, Cu-C, Cu-Si and Cu-Ge nanoparticle paste models.

The model construction was completed in two steps. First, individual spherical Cu nanoparticles with a face-centered cubic (FCC) crystal structure were generated using the lattice parameter of bulk copper [32] with a particle radius of 22.5 Å. Subsequently, a 2 × 2 assembly of Cu nanoparticles was constructed using the replicate command and placed in a periodic simulation box (Lx × Ly × Lz = 84.3 Å × 84.6 Å × 89.6 Å), forming a three-dimensional skeleton composed purely of Cu nanoparticles (23,192 Cu atoms). For the composite systems, after arranging the particles, a single monolayer sheet of graphene (2800 atoms), silicene (1144 atoms), or germanene (985 atoms) was inserted into the inter-particle region. Each sheet (approximately 84.3 Å × 84.6 Å) was placed parallel to the particle mid-plane with an initial Cu–sheet spacing of 3 Å, yielding four initial models: pure Cu, Cu-C, Cu-Si, and Cu-Ge (Figure 1).

The interatomic interactions were described using a hybrid combination of potentials [33]: Cu-Cu interactions were modeled by the embedded-atom method (EAM) potential developed by Becker et al. [34], graphene was described by the adaptive intermolecular reactive empirical bond order (AIREBO) potential [35], and silicene and germanene were represented by the Tersoff potentials parameterized by Kumagai et al. [36] and Mahdizadeh et al. [37], respectively. The non-bonded Cu–sheet interfacial interactions were modeled using a truncated 12-6 Lennard-Jones potential. The LJ parameters (ε = 0.02578 eV and σ = 3.0825 Å), originally adopted from Ref. [38] for the Cu-C cross interaction, were also applied to the Cu-Si and Cu-Ge interfaces as an effective interfacial coupling. This preserves a unified cross-interaction strength and enables a more direct evaluation of how the intrinsic structural characteristics of graphene, silicene, and germanene affect the sintering and tensile behavior. The LJ term was applied exclusively to Cu–sheet crosspairs and truncated at rc = 2.5σ (7.706 Å), implemented via lj/cut in LAMMPS. Within the hybrid potential framework, EAM was applied only to Cu-Cu pairs, AIREBO/Tersoff only to intra-sheet interactions, and the Cu–sheet coupling was described solely by the LJ term, thereby avoiding double counting of interfacial forces.

The sintering and tensile processes were sequentially performed within the same computational framework. The entire simulation consisted of six consecutive thermal and mechanical stages, with a total duration of approximately 1.3 ns: (1) A pre-equilibration stage at 300 K and 0.1 MPa for 50 ps under the NPT ensemble to achieve initial system equilibrium. The total potential energy during this stage was monitored for all four systems, showing a rapid initial decrease followed by minor fluctuations around a stable average. While the absolute energy levels differed slightly among the systems, each reached equilibrium within 50 ps. (2) A heating and pressurization stage, where the temperature was increased from 300 K to the target sintering temperature (Tsint = 500, 550, 600, and 650 K) and the external pressure was raised linearly from 0.1 MPa to 600 MPa over 300 ps. The MSD of Cu atoms was calculated specifically during this stage, ensuring that the MSD reflected the actual sintering process without interference from the initial relaxation. (3) An isothermal–isobaric sintering stage, maintained at Tsint and 600 MPa for 300 ps to facilitate interfacial rearrangement and atomic diffusion. (4) A cooling and depressurization stage, returning the system to 300 K and 0.1 MPa over 300 ps. (5) A re-equilibration stage at ambient conditions (300 K, 0.1 MPa) for another 300 ps, allowing the cooled structure to fully relax. (6) An MD-accessible strain rate stage at 300 K, where a uniaxial strain rate of 0.01 ps^−1^ was applied along the x-direction under lateral NPT control for 40 ps, yielding stress–strain curves that reveal the mechanical response of the sintered structures. In addition, all reported quantities were obtained from time-averaging over the steady-state interval of the production trajectory. The intrinsic fluctuations were assessed by block-averaging the steady-state data, and the comparative trends were found to be insensitive to the specific averaging window.

The sintering and tensile parameters were selected by considering both the physical evolution of nanoparticle sintering and the time scale limitation of molecular dynamics simulations. The multi-stage thermal–mechanical schedule was arranged to allow sequential equilibration, diffusion-assisted neck growth, structural relaxation after cooling, and subsequent tensile loading. The selected sintering temperatures were used to compare temperature-dependent differences in diffusion and mechanical response within a unified framework, whereas the tensile strain rate was chosen in a commonly adopted MD range to ensure stable calculation and clear resolution of the stress–strain behavior. This parameter selection strategy is consistent with previous MD studies on nanoparticle sintering and sintered nanostructures under mechanical loading.

**Figure 2:**
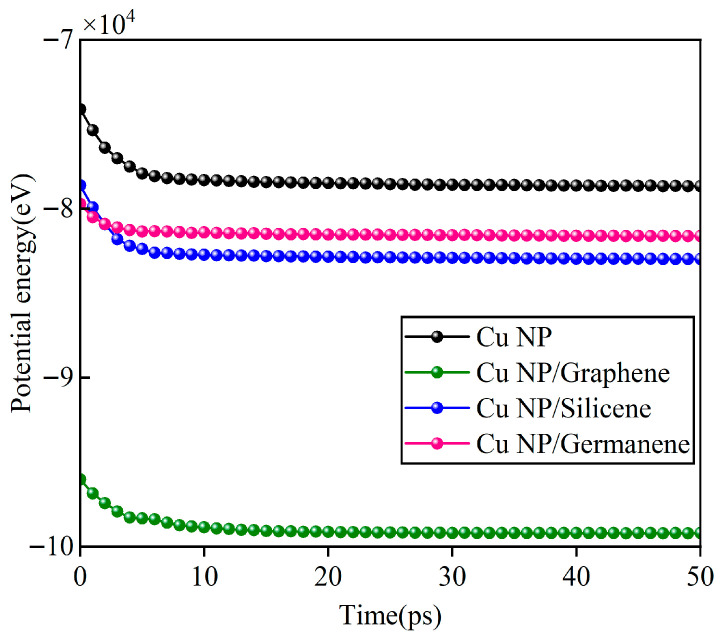
Temporal evolution of the total potential energy for the four nanoparticle paste models.

As shown in Figure 2, the total potential energy decreases rapidly at the beginning and then reaches a stable plateau with only small fluctuations, indicating that the four paste models have been adequately relaxed. This energetic stabilization provides a consistent baseline for the following sintering-stage analyses.

For data acquisition during the sintering stage, the MSD and RDF of Cu atoms were calculated to quantify diffusion intensity and characterize local coordination. Common Neighbor Analysis (CNA) was employed to identify local crystal structures (e.g., FCC, HCP), enabling assessment of structural evolution in both neck regions and particle interiors. During the tensile stage, the volume-averaged stress of the system was recorded to construct stress–strain curves, while the dislocation extraction algorithm (DXA) was applied to determine dislocation length and type. Additionally, the atomic von Mises stress field was output to analyze stress concentration zones and failure modes across different systems. These datasets enable a systematic comparison of the effect of graphene, silicene, and germanene doping on the sintering behavior and mechanical properties of copper nano-solder pastes.

## 3. Results and Discussion

### 3.1. Sintering and Strengthening Mechanism

**Figure 3:**
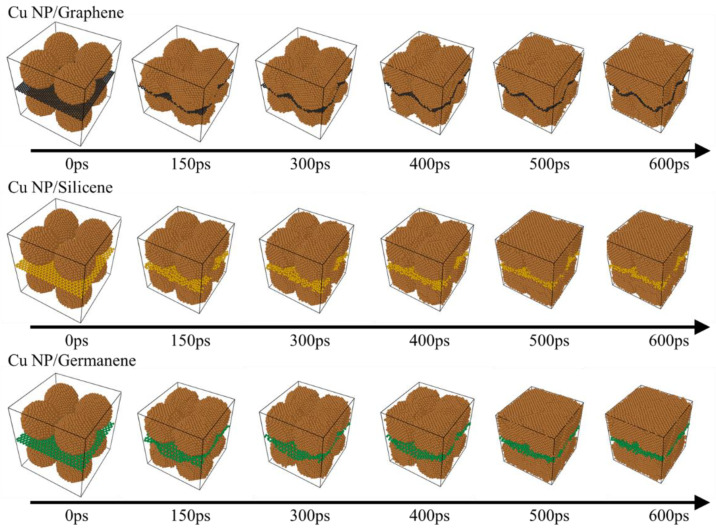
Microstructural evolution of Cu-C, Cu-Si and Cu-Ge composite pastes during sintering at 500 K.

Figure 3 displays the representative microstructural evolution of the Cu-C, Cu-Si, and Cu-Ge composite pastes from initial configuration to the end of sintering at 500 K. All three systems started with identical copper nanoparticle arrangements and 2D sheet positions. Over time, the copper nanoparticles gradually lost their polyhedral contours, with interparticle necks thickening and coalescing, eventually forming a continuous metallic network—a common characteristic across all systems. However, the temporal sequence reveals distinct differences in sintering kinetics: In the Cu-Ge system, the copper particles underwent extensive, near-melting-like rearrangement within a relatively short time, with particle boundaries rapidly blurring and pores near the germanene being filled early. The Cu-Si system exhibited a moderate densification rate, where interparticle interfaces remained discernible, yet interconnected channels had formed. In contrast, the Cu-C system showed the slowest neck formation, with most copper particles retaining relatively clear outlines, even at the end of sintering.

This observed progression can be understood by considering the intrinsic material properties of the 2D fillers. Germanene possesses relatively low in-plane stiffness, making it prone to undulations. These deformations provide additional contact opportunities and rearrangement space for surrounding copper atoms, thereby facilitating rapid particle coalescence. Silicene, with intermediate in-plane stiffness, promotes and partially constrains copper mobility. Graphene, characterized by the highest in-plane stiffness and a stable hexagonal lattice, resists significant bending during copper particle movement, maintaining a flatter interface. This largely restricts copper deformation to localized regions, prolongs the sintering process, and results in the most preserved particle morphology in the final structure.

**Figure 4:**
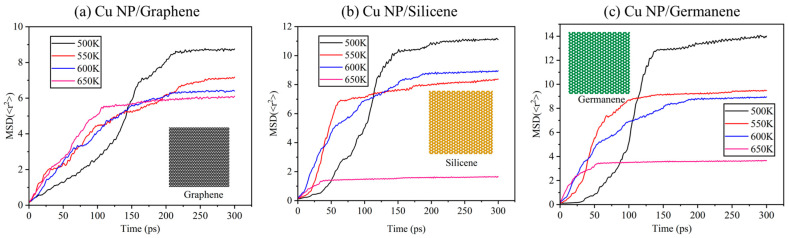
MSD of Cu atoms in (**a**) Cu-C, (**b**) Cu-Si and (**c**) Cu-Ge composite pastes at different sintering temperatures.

Figure 4 presents the temporal evolution of the MSD of Cu atoms in the three composite pastes at different temperatures. For each system, the MSD increases rapidly during the initial sintering stage before gradually plateauing, indicating a transition from substantial atomic rearrangement to predominantly localized vibrations at the given temperature. Comparing different temperatures reveals that higher sintering temperatures significantly shorten the time required to reach the plateau stage, while the plateau MSD values generally decrease. This suggests that at elevated temperatures, copper atoms undergo more intensive rearrangement and flow in the early stages but become more tightly constrained within the sintered network by the end of the observation period, resulting in smaller long-range displacements. This behavior aligns with the previously observed structural features of enhanced neck growth and reduced porosity at higher temperatures.

When comparing the three composite systems at identical temperatures, discernible differences emerge in the plateau regions of the MSD curves. At most temperature points, the Cu-Ge system exhibits the highest equilibrium MSD, the Cu-C system shows the lowest, and the Cu-Si system falls between them. These variations can be rationalized through the corresponding sintering morphologies: In the Cu-Ge system, the pronounced undulations and local folding of the germanene layer during sintering create residual space for local atomic rearrangements even after structural stabilization, maintaining a relatively high MSD. In the graphene-reinforced system, the high stiffness of the carbon skeleton and flat interfaces lead to earlier fixation of neck regions, strongly restricting long-term copper displacement and yielding the lowest equilibrium MSD. The silicene system demonstrates intermediate characteristics, consistent with its moderate MSD values. Overall, the MSD profiles reflect the varying degrees of constraint imposed by different 2D fillers on copper atomic mobility after sintering, corroborating the observed differences in densification behavior and neck morphology.

It should be noted that the present simulations were performed using ideal defect-free monolayers. In practical synthesis, however, point defects and local lattice imperfections are often unavoidable and may affect local stiffness, surface corrugation, and interfacial contact with Cu nanoparticles, thereby influencing the quantitative results. Nevertheless, the present models provide a controlled basis for revealing the intrinsic comparative behavior of graphene, silicene, and germanene under unified conditions.

**Figure 5:**
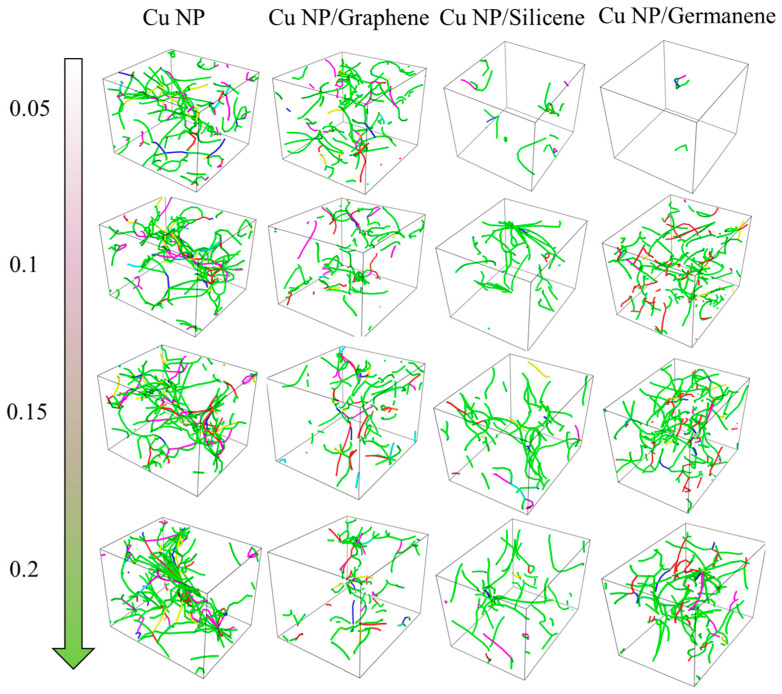
Dislocation configurations of pure Cu, Cu-C, Cu-Si and Cu-Ge pastes during tensile loading at 300 K.

Figure 5 presents the dislocation structures of the pure Cu system and the three composite systems during tensile testing at 300 K with a strain rate of 0.01 ps^−1^. The images correspond to different tensile strain levels, arranged from top to bottom. At relatively low strain levels, the pure Cu system already exhibits the formation of continuous dislocation lines, with preliminary dislocation networks being visible in local regions. In the Cu-Ge composite system, significantly fewer dislocations are observed within the Cu regions; most dislocations concentrate near the Ge sheets, while large dislocation-sparse volumes remain inside the particles. The dislocation distribution in the Cu-C composite system more closely resembles that of pure Cu. This can primarily be attributed to the fact that during initial stretching, the Ge sheets in the Cu-Ge system accommodate part of the deformation through their own stretching and local undulations, resulting in a slower increase in shear stress within the Cu regions. In contrast, the stiffer graphene sheets undergo minimal contour changes, transmitting external loads to the metal phase earlier, and thus triggering substantial dislocation activity in the Cu phase of the Cu-C system at smaller strains.

As the strain increases to higher levels, the dislocation distribution undergoes significant changes. The relative sparsity of dislocation lines follows the order: graphene composite system > Si composite system > Ge composite system ≈ pure Cu. In other words, at high strain stages, deformation in the Cu-Ge and pure Cu systems is primarily accommodated by dislocation activity within the Cu phase itself. In the Cu-Si composite system, the Si phase still bears a portion of the strain, with some stress relaxation occurring near the interfaces, maintaining a moderate dislocation density in the Cu regions. Conversely, in the graphene-reinforced system, a considerable proportion of the deformation is still carried by the stretching and bending of the graphene layer, along with interfacial relative displacement, resulting in a relatively lower accumulation rate of dislocations within the Cu phase. This indicates that at equivalent strain levels, the Cu-Ge and pure Cu systems rely more on extensive dislocation motion within the matrix, while the Cu-C system tends to distribute deformation through the 2D skeleton and interfacial regions, thereby delaying the generation and propagation of large-scale dislocations in the Cu regions.

**Figure 6:**
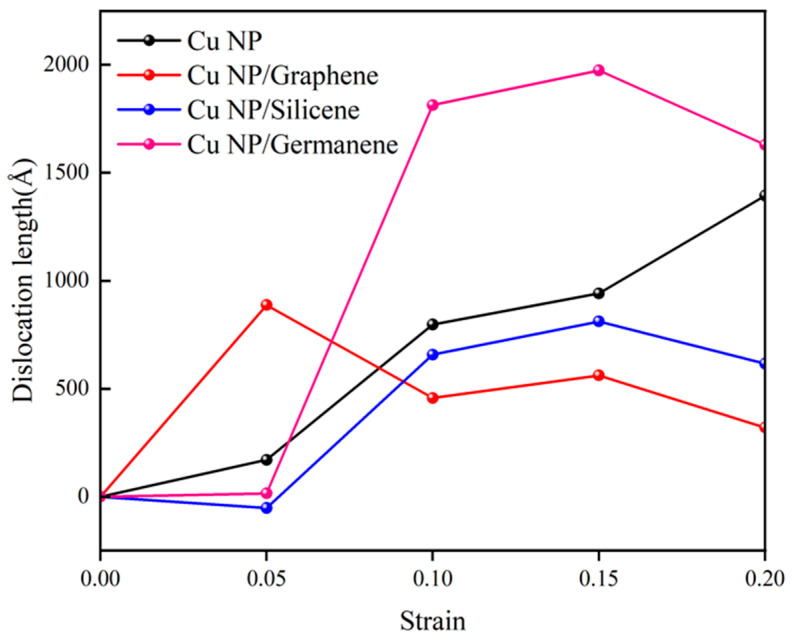
Strain–dislocation length curves for pure Cu, Cu-C, Cu-Si and Cu-Ge pastes at 300 K.

Figure 6 shows the relationship between strain and total dislocation length for the four systems under tensile testing at 300 K with a strain rate of 0.01 ps^−1^. The dislocation length was quantified using the dislocation extraction algorithm (DXA) and normalized by subtracting the initial value at zero strain; thus, the curves represent the generated or extended dislocation content during tensile deformation. Overall, the curves for pure Cu, Cu-Si, and Cu-Ge generally rise with increasing strain, indicating that plastic deformation in these three systems is primarily accommodated by a continuous increase in dislocations. In contrast, the Cu-C curve reaches a relatively high level at small strains, followed by a subsequent decrease in dislocation length with further strain.

In terms of variation magnitude, the total dislocation length in pure Cu increases approximately sevenfold as the strain rises from 0.05 to 0.20, demonstrating continuous accumulation. For Cu-Ge, dislocations are nearly negligible at a strain of 0.05 but rapidly increase to around 1800–2000 Å within the 0.10–0.15 strain range over two orders of magnitude higher than the initial value, representing the highest level among all systems. The dislocation length in Cu-C reaches nearly 900 Å at 0.05 strain and then decreases to approximately 320 Å with further strain, suggesting that a large number of dislocations generated early in the deformation are subsequently annihilated or reorganized into localized regions. This trend aligns with the dislocation morphology analysis in the previous section: at low strains, the Cu-C system exhibits the densest dislocation distribution, whereas Cu-Ge and Cu-Si show limited dislocation accumulation; as strain further increases, dislocations in Cu-Ge rapidly propagate throughout particles and neck regions, pure Cu follows closely, Cu-Si maintains a moderate level, while Cu-C experiences slowed growth in total dislocation length due to annihilation and localized reorganization.

The evolution of total dislocation length is closely associated with the strain-accommodation behavior of the sintered structures. In general, a sustained increase in dislocation length with tensile strain suggests that plastic deformation can continue to be accommodated through dislocation nucleation, multiplication, and propagation in the Cu matrix. This helps redistribute local stress and suppresses early strain localization, which is favorable for maintaining mechanical integrity during deformation. In the present study, the Cu-Ge system exhibits the most pronounced increase in dislocation length at medium-to-high strain levels, indicating a stronger ability to accommodate deformation through continued dislocation activity. This trend is consistent with its higher tensile strength. In contrast, although the Cu-C system also shows an enhanced mechanical response, its deformation is more strongly shared by the carbon sheet and the interface region, so the contribution from continued dislocation accumulation in the Cu phase becomes less dominant at larger strains.

### 3.2. Impact of the Different Sintering Parameters on the Mechanical Properties

In the present simulations, the sintering holding time was fixed at 300 ps, whereas the sintering temperature was varied systematically. This setting was adopted to isolate the effect of temperature on the evolution of the sintered structure and its subsequent mechanical behavior. It should be noted that, because molecular dynamics is inherently limited to the ps-ns time scale, the present holding time does not correspond directly to macroscopic experimental sintering durations. Instead, it represents an MD-accessible observation window for capturing the early-stage atomistic processes of nanoscale sintering, including interfacial rearrangement, diffusion-assisted neck growth, and local pore shrinkage. Within this fixed holding time, increasing temperatures still produce marked differences in the final mechanical response, because a higher temperature accelerates atomic diffusion and facilitates more effective structural evolution before cooling. Therefore, the results in this section should be understood as a comparison of temperature effects under a constant MD-accessible sintering time, rather than a full time–temperature equivalence analysis.

**Figure 7:**
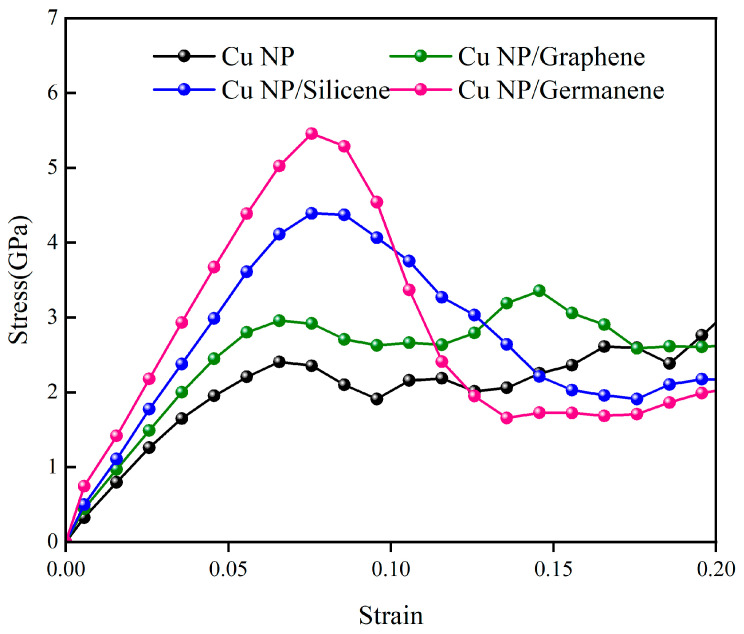
Tensile stress–strain curves of pure Cu paste and Cu-C, Cu-Si, Cu-Ge composite pastes at 300 K.

Figure 7 presents the tensile stress–strain curves at 300 K for the pure Cu paste and the Cu-C, Cu-Si, and Cu-Ge composite pastes after sintering at 600 K and subsequent cooling to 300 K under identical conditions and strain rates. In all four systems, the stress first increases with strain and then enters a softening regime after the peak. The curves do not exhibit a long linear elastic segment as in dense bulk metals; instead, noticeable curvature appears already at small strains, which reflects the presence of pores, neck regions, and a high density of crystal defects in the sintered networks.

Using yield strength as an indicator of load-bearing capacity, the pure Cu solder paste exhibits a value of 2.41 GPa, which is the lowest among the four systems. The introduction of graphene increases the yield strength to 2.96 GPa, corresponding to an improvement of about 23% relative to pure Cu. Doping with silicene and germanene further enhances the yield strength to 4.39 GPa and 5.46 GPa, respectively, representing increases of approximately 82% and 127% compared with pure Cu. Under identical sintering conditions, all three two-dimensional fillers therefore strengthen the sintered structure, with graphene providing moderate reinforcement, while silicene and germanene lead to much more pronounced strengthening.

Combined with the previous analysis of dislocation structures, the Cu-Ge system exhibits extensive dislocation accumulation and wider high-stress regions at large strains, consistent with its superior plastic accommodation and load-bearing capability. In the Cu-C system, part of the deformation is still absorbed by the graphene sheet and interfacial regions, which limits dislocation build-up in the Cu phase and results in a more moderate increase in yield strength compared with the Cu-Si and Cu-Ge pastes, while still outperforming the pure Cu paste.

**Figure 8:**
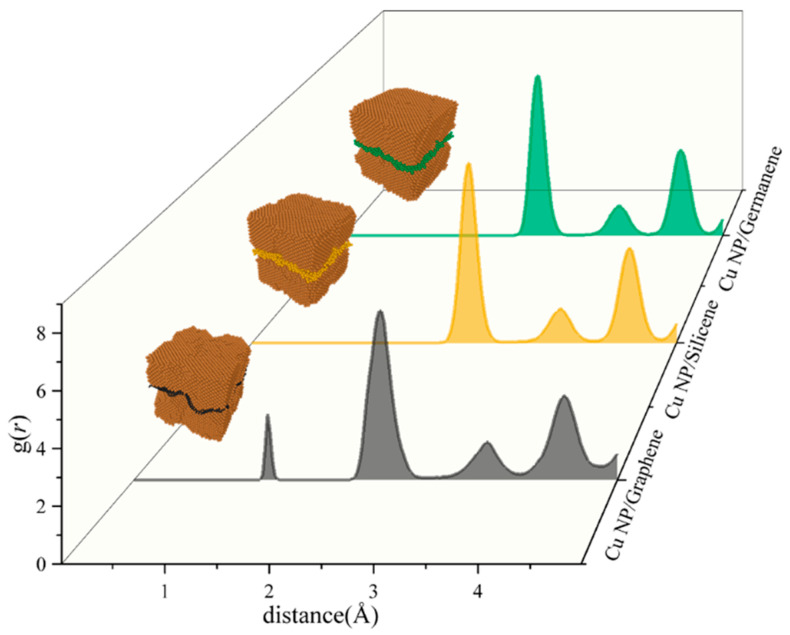
RDFs at the Cu-C, Cu-Si and Cu-Ge interfaces.

Despite clear differences in strength levels, the stress–strain curves of both pure Cu and the three composite pastes lack the distinct linear elastic region that is typically observed in dense bulk metals, instead exhibiting noticeable curvature and nonlinearity from the early stages of deformation. Figure 8 shows the RDF at the interfaces between the three types of 2D fillers and Cu nanoparticles. In all three systems, the primary RDF peaks between the filler atoms and Cu atoms are located near 2.5 Å, indicating a consistent dominant atomic interaction distance across the interfaces. The overall RDF profiles are broadly similar, with relatively low and smooth primary peak intensities, suggesting a low probability of coordinated atomic packing at the interfaces and generally weak interfacial interactions between the fillers and copper.

This weak interfacial bonding results in minimal strong constraint from the 2D fillers on the deformation behavior of the Cu nanoparticles in the sintered composites. Consequently, the dominant mechanisms in the initial deformation stage are the inhomogeneous rearrangement of surface atoms within the Cu nanoparticles themselves and the non-uniform stretching of interparticle bonds. These mechanisms collectively lead to the absence of a typical linear elastic segment in the stress–strain curves of all systems, which instead display curved profiles right from the initial loading stage.

**Figure 9:**
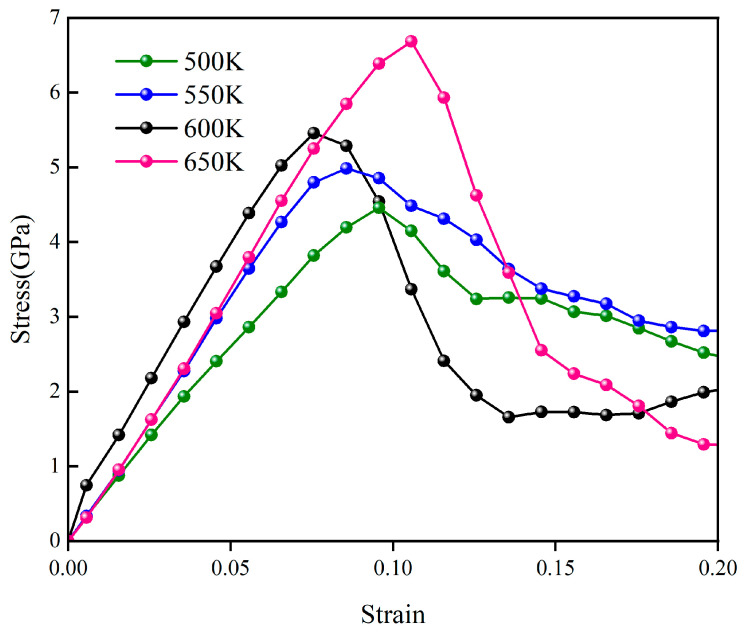
Tensile stress–strain curves of Cu-Ge pastes sintered at different temperatures.

Figure 9 further presents the stress–strain curves of the Cu-Ge composite when sintered at different temperatures. All four curves share a similar profile: the stress first rises with strain, reaches a single peak at an engineering strain of about 0.08–0.11, and then enters a marked softening stage. This response indicates that the porous metallic network formed after sintering experiences elastic deformation and early local plastic rearrangement during initial loading, followed by necking and damage propagation once the maximum load-bearing capacity is reached.

With increasing sintering temperature, the stress–strain response becomes progressively stronger. The peak stress increases from about 4.5 GPa for the 500 K sample to roughly 5.0 GPa, 5.5 GPa, and 6.7 GPa for the 550 K, 600 K, and 650 K samples, respectively, showing a monotonic rise in peak tensile strength (maximum stress). This trend reflects the temperature-enhanced sintering kinetics: higher temperatures promote neck growth between particles, reduce the residual porosity, and enlarge the effective load-bearing cross-section, so that the tensile strength is governed mainly by densification rather than by coarsening within the examined temperature window.

The post-peak behavior also changes with sintering temperature. The 650 K curve exhibits the steepest stress drop after the peak, whereas the specimens sintered at lower temperatures retain comparatively higher residual stresses in the softening regime. This behavior can be attributed to the coarser neck structures formed at elevated temperatures, which provide stronger interparticle connections but also cause more concentrated plastic deformation and faster damage accumulation. As a result, the Cu-Ge composite sintered at 650 K combines the highest peak strength with a sharper loss of load-bearing capacity once necking and fracture initiate.

**Figure 10:**
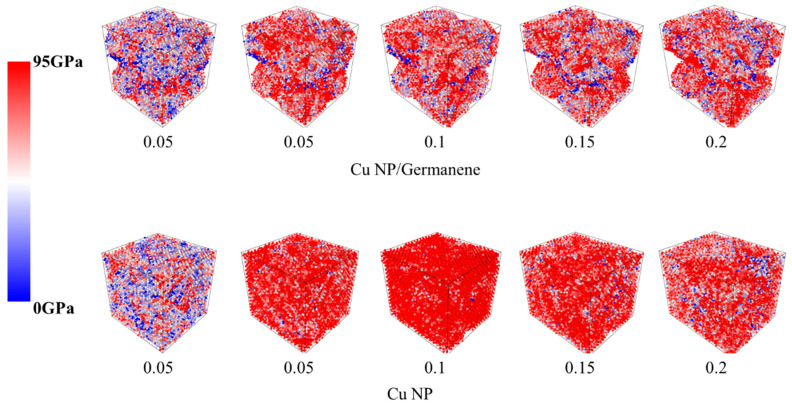
Atomic stress maps of Cu NP/Germanene sintered composite paste and Cu NP paste in uniaxial tensile process along with the *X*-axis.

Figure 10 compares the atomic von Mises stress distributions in the Cu-Ge composite and pure Cu pastes under identical temperature but different tensile strains, with the color scale ranging from blue to red corresponding to 0–95 GPa. In the Cu-Ge system, at relatively low strain, high-stress atoms appear in scattered patches, primarily concentrated at particle necks and regions adjacent to the germanene sheets, while a significant volume of the bulk remains at low-to-medium stress levels. As strain increases, these high-stress zones expand along particle contact paths and around the germanene sheets. Throughout the deformation, a considerable fraction of low- and medium-stress regions persists, indicating that the interfaces provide a buffering and regulating effect, enabling multi-path load transfer and thereby supporting higher macroscopic load-bearing capacity. In contrast, the stress evolution in the pure Cu paste tends toward a globally high stress state. At intermediate strain, most of the cross-section is occupied by high-stress atoms, with only isolated low-stress spots near a few pores, reflecting a near-saturated load distribution within the metallic network. At higher strains, new medium- and low-stress zones emerge around the primary necking region, consistent with the rapid post-peak softening in the stress–strain curve. Once the main neck becomes unstable and begins to fail, the surrounding area unloads rapidly, forming a localized stress relaxation zone.

## 4. Conclusions

This study employed MD simulations to conduct a comparative analysis of the behavior of pure copper and copper nanoparticle solder pastes doped with graphene, silicene, and germanene under various sintering temperatures and tensile loading conditions. The main conclusions are as follows:

1. All three composite pastes formed continuous copper networks under identical sintering conditions, yet exhibited distinct densification kinetics: neck formation and pore closure were fastest with germanene, intermediate with silicene, and slowest with graphene. Increasing the sintering temperature accelerated early atomic rearrangement, resulting in thicker necks and fewer residual pores.
2. MSD and RDF results indicate that all Cu-2D interfaces exhibit a short-range ordered but long-range disordered structure. The resultant localized rearrangement of copper atoms near these interfaces effectively dissipates stress during early loading, accounting for the absence of a distinct linear elastic regime in the stress–strain curves of all pastes.
3. Tensile tests at 300 K on structures sintered at 600 K showed that the yield strength of pure Cu was 2.41 GPa. Graphene increased the yield strength to 2.96 GPa, while silicene and germanene further raised it to 4.39 GPa and 5.46 GPa, corresponding to enhancements of about 23%, 82%, and 127% over pure Cu.
4. For the Cu-Ge composite, raising the sintering temperature from 500 K to 650 K caused a monotonic increase in peak tensile strength, with the 650 K sample reaching the highest value. The stronger specimens, however, exhibited a steeper post-necking stress drop, indicating enhanced strain localization and a trade-off between strength and damage tolerance.

In summary, both the type of group IV 2D material and the sintering temperature can effectively tailor the strength and deformation behavior of copper nano-solder paste interconnection layers. These conclusions should be interpreted within the scope of a fixed MD-accessible sintering holding time and an idealized material model. Within this framework, an increasing sintering temperature leads to clear changes in structural evolution and final mechanical response, while the superior performance of the Cu-Ge system is attributed to its more sustained dislocation activity during deformation. Although longer holding times, impurities, oxidation, and more complex interfacial chemistry may influence the quantitative results, the comparative trends and mechanistic insights obtained here provide a useful basis for understanding the strengthening role of group IV 2D fillers in copper nano-solder pastes and for guiding future experimental design and process optimization.

## Data Availability

The original contributions presented in this study are included in the article. Further inquiries can be directed to the corresponding author.
